# Protective effects of delphinidin against H_2_O_2_–induced oxidative injuries in human retinal pigment epithelial cells

**DOI:** 10.1042/BSR20190689

**Published:** 2019-08-15

**Authors:** Timin Ni, Wanju Yang, Yiqiao Xing

**Affiliations:** 1 Department of Ophthalmology, Renmin Hospital of Wuhan University, Wuhan 430060, China; 2 Department of Ophthalmology, Wuhan Central Hospital, Wuhan 430014, China

**Keywords:** Age-related macular degeneration, Antioxidant, Apoptosis, Delphinidin, Human retinal pigment epithelial cells, Oxidative stress

## Abstract

Age-related macular degeneration (AMD) is now one of the leading causes of blindness in the elderly population and oxidative stress-induced damage to retinal pigment epithelial (RPE) cells occurs as part of the pathogenesis of AMD. In the present study, we evaluated the protective effect of delphinidin (2-(3,4,5-trihydroxyphenyl) chromenylium-3,5,7-triol) against hydrogen peroxide (H_2_O_2_)-induced toxicity in human ARPE-19 cells and its molecular mechanism. MTT (3-(4,5-dimethylthiazol-2-yl)-2,5-diphenyltetrazolium bromide) assay and flow cytometry demonstrated that pretreatment of ARPE-19 cells with delphinidin (25, 50, and 100 μg/ml) significantly increased cell viability and reduced the apoptosis from H_2_O_2_ (0.5 mM)-induced oxidative stress in a concentration-dependent manner, which was achieved by the inhibition of Bax, cytochrome *c*, and caspase-3 protein expression and enhancement of Bcl-2 protein. The same tendency was observed in ARPE-19 cells pre-treated with 15 mM of N-acetylcysteine (NAC) before the addition of H_2_O_2_. Furthermore, pre-incubation of ARPE-19 cells with delphinidin markedly inhibited the intracellular reactive oxygen species (ROS) generation and Nox1 protein expression induced by H_2_O_2_. Moreover, the decreased antioxidant enzymes activities of superoxide dismutase (SOD), catalase (CAT), and glutathione-peroxidase (GSH-PX) and elevated (MDA) level in H_2_O_2_-treated cells were reversed to the normal standard by the addition of delphinidin, which was regulated by increasing nuclear Nrf2 protein expression in ARPE-19 cells. Our results suggest that delphinidin effectively protects human ARPE-19 cells from H_2_O_2_-induced oxidative damage via anti-apoptotic and antioxidant effects.

## Introduction

Age-related macular degeneration (AMD) is the most devastating retinal disease, which causes blindness among the elderly population throughout the world [1], and currently there are still no curative methods available for this disease [2,3]. Although the exact pathogenesis of AMD remains largely unknown, oxidative stress-induced dysfunction of retinal pigment epithelium (RPE) cells leading to secondary photoreceptor loss is suggested as a pathological cause in the early AMD stage [4,5]. The RPE is a monolayer of pigmented cells located in the outer layer of the retina to form a part of the blood–retina barrier [6] and secretes a variety of growth factors to maintain the regeneration and repair of photoreceptor cells [7]. The retina is a tissue with the highest oxygen consumption in comparison with other tissues, indicating that the retinal RPE cells are more vulnerable to the damage by oxidative stress, particularly from reactive oxygen species (ROS) [8,9]. Growing evidence prove that ROS-induced irreversible damage to RPE cells is thought to be an early event in AMD [10]. Accordingly, the extensive degeneration of RPE with increasing age causes the death of photoreceptor cells thereby leading to vision loss in AMD patients [11]. As such, various approaches to limit oxidative stress in the RPE cells may be an effective strategy for the amelioration of early AMD.

A large number of studies suggest naturally occurring antioxidant agents can ameliorate age-related changes associated with oxidative damage in RPE cells. For instance, blueberry anthocyanins could inhibit the induction and progression of AMD through antioxidant mechanisms [12]. Lutein and zeaxanthin supplementation can contribute to the prevention of AMD, not only by increasing the macular pigment density, but also by protecting RPE cells against oxidative stress [13]. Therefore, discovery of effective dietary countermeasures, especially antioxidants, to delay or slow down the progression of this disease has been a topic of increasing interest. Anthocyanidins are naturally occurring organic flavonoids that belong to a polyphenol group and commonly exist in fruits or vegetables as six components including cyanidin (50%), delphinidin (12%), malvidin (12%), pelargonidin (12%), peonidin (7%), and petunidin (7%) [14]. Among the six anthocyanidins, delphinidin (2-(3,4,5-trihydroxyphenyl) chromenylium-3,5,7-triol) exerts the strongest antioxidative efficiency of all anthocyanidins in the human diet due to the presence of a large number of hydroxyl groups in its structure which enhance reactivity [15,16] and is known to be predominantly in many pigmented fruits and vegetables such as pomegranate, carrot, eggplants, berries, and red onions [17]. In addition, delphinidin exhibits anti-inflammatory, antiproliferative, and antitumor activities in a variety of cancer cells [18]. On the basis of its antioxidant effects, delphinidin can protect human HaCaT keratinocytes and mouse skin from UV-induced oxidative stress and apoptosis via down-regulation of UV-induced PTGS2 expression by inhibiting MAPKK4 and PI3K [19,20]. Also, the inhibition of PI3K/Akt pathway was involved in the protective effect of delphinidin on human embryonic stem cells against hypoxia-induced apoptosis and oxidative stress [21]. However, the protective effects of delphinidin on retinal epithelial cells exposed to hydrogen peroxide (H_2_O_2_) are still unknown. Therefore, in the present study, we aim to investigate the protective effect of delphinidin against H_2_O_2_ toxicity in ARPE-19 cells, a widely used *in vitro* model of human RPE, and subsequently explore the mechanisms underlying the antioxidant effect of delphinidin.

## Materials and methods

### Materials and chemicals

**Figure 1:**
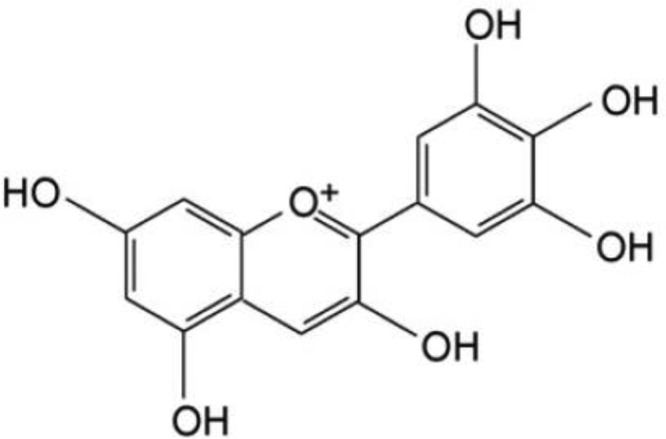
Chemical structure of delphinidin

Dulbecco’s Modified Eagle’s Medium (DMEM), penicillin/streptomycin, and certified fetal bovine serum were purchased from Invitrogen (Carlsbad, CA, U.S.A.). Delphinidin (Figure 1), fetal bovine serum, penicillin, streptomycin, amphotericin B, 3-(4,5-dimethylthiazol-2-yl)-2,5-diphenyltetrazolium bromide (MTT), 2′,7′-dichlorofluorescin diacetate (DCFH-DA), N-acetylcysteine (NAC), and dimethylsulfoxide (DMSO) were purchased from Sigma–Aldrich, Inc (St. Louis, MO, U.S.A.). The Annexin V−Fluorescein Isothiocyanate (FITC)/Propidium Iodide (PI) Apoptosis Detection Kit was obtained from BD Biosciences (CA, U.S.A.). Superoxide dismutase (SOD), catalase (CAT), glutathione-peroxidase (GSH-PX) and malondialdehyde (MDA) assay kits were obtained from Xinzhetianyou Biotechnology Company, Ltd. (Beijing, China). BCA protein assay kit was from Meixuan Biological Science and Technology, Ltd. (Shanghai, China). Primary antibodies including anti-Bcl-2, anti-Bax, anti-cleaved caspase-3, anti-cytochrome *c*, anti-Nrf2, anti-Nox1 anti-β-actin, and horseradish peroxidase–conjugated secondary antibodies were purchased from Santa Cruz Biotechnology (Santa Cruz, CA, U.S.A.). All the chemicals and reagents were of analytical grade.

### Cell line and culture

The human retinal pigment epithelium cell line (ARPE-19 cells) was purchased from Shanghai Institute of Cell Biology, Chinese Academy of Sciences (Shanghai, China) and was routinely maintained in DMEM/F-12 medium, supplemented with 10% fetal bovine serum, 100 U/ml penicillin, and 100 μg/ ml streptomycin in a 37°C incubator under a humidified atmosphere (95% air, 5% CO_2_). These cells were passaged by 0.25% Trypsin/EDTA every 3–4 days and used within the first ten passages.

### MTT assay

Cell viability was checked using MTT assay. In brief, to create an H_2_O_2_-induced RPE cell injury model, ARPE-19 cells (1 × 10^5^ cells/well) were exposed with H_2_O_2_ (0–0.5 mM) for 4 h and the effect of delphinidin on cell viability of ARPE-19 cells was also evaluated with different concentrations (12.5–200 μg/ml) for 24 h. To examine the protective effects of delphinidin against H_2_O_2_-induced toxicity, cultured ARPE-19 cells were pre-treated with different concentrations of delphinidin for 24 h, followed by a 4-h exposure of H_2_O_2_ (0.5 mM). After various treatments, 10 μl of MTT solution (5 mg/ml) was added to each well and the cells were incubated for another 4 h at 37°C. Then the medium was aspirated from each well and 100 μl DMSO was added to dissolve formazan crystals by gentle agitation for 10 min. Finally, the absorption was evaluated spectrophotometrically at a wavelength of 490 nm using a microplate reader (BioTek, Winooski, VT, U.S.A.). Delphinidin was diluted in DMSO which was used for vehicle control in all assays. The final concentrationof DMSO in experiments never exceeded 0.1%. All experiments were performed in triplicates. The relative cell viability was defined as percentage of the controls, which was set as 100%.

### Flow cytometry analysis for cell apoptosis

Cell apoptosis was examined by Annexin V-FITC/PI Apoptosis Detection Kit according to the manufacturer’s protocol. ARPE-19 cells were grown in a six-well plate at a density of 2 × 10^5^ cells/well and treated with or without delphinidin for 24 h, before treatment with 0.5 mM H_2_O_2_ for 4 h. After that, the cultured cells in all groups were washed twice with ice-cold PBS, resuspended in 300 μl binding buffer, and stained with 10 μl of Annexin V-FITC stock and 10 μl of PI in the dark for 20 min. Immediately, the stained cells were analyzed with a FACScan Calibur Flow Cytometer (BD Bioscience, NJ, U.S.A.), and the percentage of apoptotic cells was calculated by CellQuest software (Becton–Dickinson, CA, U.S.A.). The results were expressed as the percentage of Annexin V-stained cells and all experiments were performed in triplicate. Morphological changes were photographed under a phase contrast microscope (Olympus, Tokyo, Japan).

### Intracellular ROS measurement

The level of intracellular ROS was quantified using ROS detection fluorescent probe DCFH-DA, which is oxidized by ROS in viable cells to 2′,7′-dichlorodihydrofluorescein (DCF). Briefly, the cells treated as described before were harvested and incubated with 10 μM of DCFH-DA in the dark for 30 min at 37°C, and then detached with trypsin/EDTA, washed, and re-suspended in PBS (1 × 10^6^ cells/ml). The intracellular fluorescence intensity was immediately recorded on flow cytometry at excitation and emission wavelengths of 488 and 525 nm and the images were taken by fluorescence microscope. The results of the ROS release were expressed as the fluorescence intensity of DCF with respect to that of the control. Ten thousand cells were analyzed in each sample and all experiments were performed in triplicate.

### Detection of oxidative and lipid oxidation biomarkers

Following treatment, the ARPE-19 cells were collected and four common oxidative biomarkers, including SOD, CAT, GSH-PX, and MDA, were used to measure the oxidative stress levels in different groups. In general, SOD, CAT, GSH-PX activity and MDA levels were detected using commercially available assay kits (Cayman Chemical Company, MI, U.S.A.), following the manufacturer’s instructions, respectively.

For SOD activity assay, the collected cells were washed thrice with PBS and centrifuged in 1500×***g*** for 5 min at 4°C. The resulting cell pellets were placed in lysis buffer [25 mM NaCl, 0.5% Triton X-100, 60 mM Tris-HCl (pH 7.4), 1 mM Na_3_VO_4_, 20 mM NaF, 10 mM Na_4_P2O_7_] containing protease inhibitor cocktail for 60 min on ice. After centrifugation (1500×***g*** for 5 min) at 4°C for 30 min, the supernatant was collected and used for the enzyme assay. Briefly, the buffer solution containing tetrazolium salt and hypoxanthine (200 μl) was mixed with 10 μl of sample and initiated by the addition of 20 μl of diluted xanthine oxidase to each well. Following gentle shaking at room temperature for 20 min, the absorbance was recorded at 460 nm using a microplate reader. A standard curve made with bovine erythrocyte SOD was used to determine enzyme activity.

For CAT activity assay, the assay method is based on H_2_O_2_ decomposition by the reaction of CAT contained in the examined samples in the presence of an optimal concentration of H_2_O_2_. In brief, the pelleted cells and the resulting supernatant were prepared as described for SOD activity assay. Each equal amount of sample (20 μl) was mixed with 100 μl of assay buffer and 30 μl of methanol, and added with 20 μl of H_2_O_2_ followed by incubation on a shaker at room temperature. After 20 min, 30 μl of potassium hydroxide and 30 μl of Purpald (chromogen) were plated to terminate the reaction. Then, the plate was incubated for another 10 min on the shaker at room temperature. Before the sample was monitored at 540 nm, 10 μl of potassium periodate was added and incubated for 5 min on shaker. A standard curve plotted with bovine liver CAT was performed to determine enzyme activity.

GSH-PX activity was determined by measuring the extent of the oxidation of nicotinamide adenine dinucleotide phosphate (NADPH). The oxidation of NADPH to NADP^+^ would lead a decrease in absorbance at 340 nm. Under this condition, GSH-PX activity is consumed and the rate of decrease in the absorbance at 340 nm is directly proportional to the GSH-PX activity in the sample. The 20-μl supernatant of sample prepared as described for SOD activity assay was treated with 100 μl of assay buffer, 50 μl of co-substrate mixture (NADPH, glutathione, and glutathione reductase) and initiated by adding 20 μl of cumene hydroperoxide. The plate was shaken for a few seconds and the absorbance was read at 340 nm. GSH-PX activity was calculated and one unit was defined as the amount of enzyme that will cause the oxidation of 1.0 nmol of NADPH to NADP^+^ per minute at 25°C.

The protein concentration of the lysate was measured by a BCA protein assay kit. Antioxidant enzymes activities in total retinal protein extraction were expressed as units per milligram protein and MDA content was expressed as nmol per milligram protein. All samples were assayed in triplicate.

### Western blot analysis

Western blot analysis was performed following the method as described previously [22]. Briefly, the ARPE-19 cells from different experimental conditions were rinsed once with ice-cold PBS and extracted using RIPA lysis buffer. Then cell lysates were centrifuged at 13000 rpm for 15 min at 4°C, and the supernatant was collected. The protein concentration was determined using a BCA protein assay kit according to the manufacturer’s instructions. Equal amounts of protein (30 μg) were separated on a 12% sodium dodecyl sulfate/polyacrylamide gel electrophoresis and transferred electrophoretically to a nitrocellulose membrane. After blocking with a 5% non-fat milk for 1 h at room temperature, the membrane was incubated with primary antibodies against cleaved caspase-3 (1:500), Bcl-2 (1:500), Bax (1:500), cytochrome *c* (1:500), Nox1 (1:500), anti-Nrf2 (1:300), and β-actin (1:1000) (Santa Cruz, CA, U.S.A.), respectively, overnight at 4°C, followed by incubation with the appropriate horseradish peroxidase–conjugated secondary antibody (1:1500), at room temperature for 1 h. The bands were visualized using an enhanced chemiluminescence (ECL) detection system (Thermo Scientific, Rockford, IL, U.S.A.) and band intensities were quantified using ImageJ software (NIH Image, Bethesda, MD, U.S.A.). All experiments were repeated three times.

### Statistical analysis

All the data are presented as the mean ± standard deviation (S.D.) of three independent experiments in triplicates. GraphPad Prism software was used to analyze the data. All statistical comparisons were carried out using one-way ANOVA followed by Tukey’s multiple comparison, with *P*<0.05 considered statistically significant.

## Results

### Delphinidin attenuates H_2_O_2_-induced cytotoxicity in ARPE-19 cells

**Figure 2:**
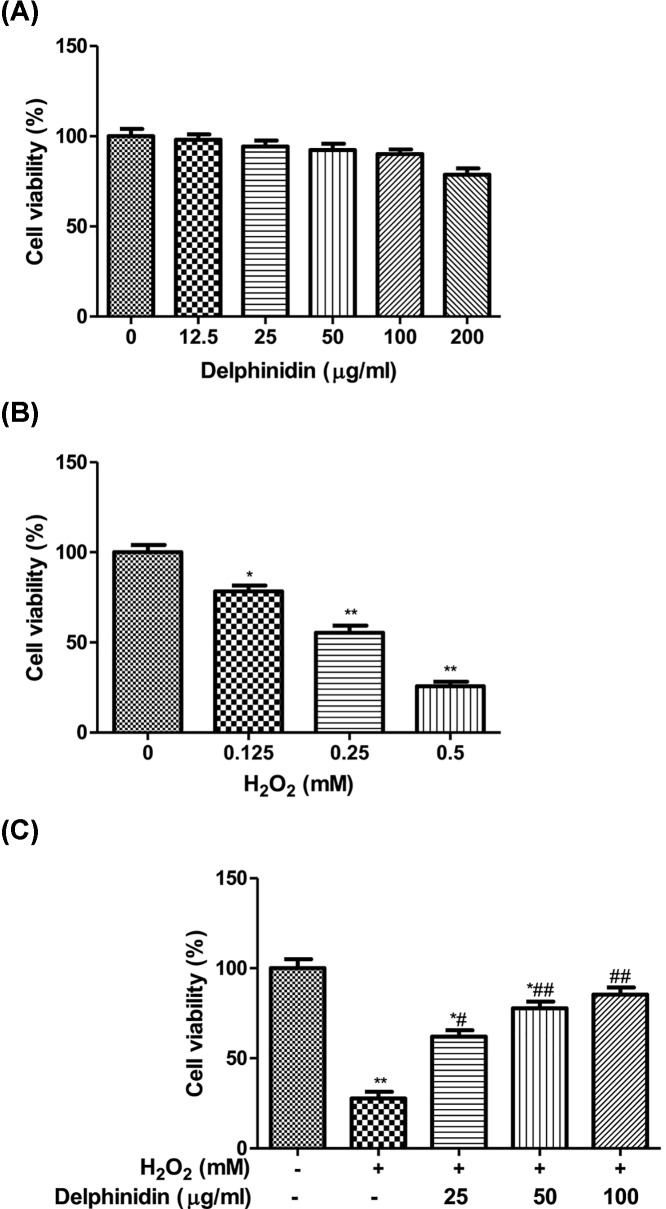
Delphinidin attenuates H_2_O_2_-induced cytotoxicity in ARPE-19 cells (**A**) Effect of delphinidin on cell viability of ARPE-19 cells. ARPE-19 cells were treated with different concentrations of delphinidin (0–200 μg/ml) for 24 h. (**B**) Effect of H_2_O_2_ on cell viability of ARPE-19 cells. ARPE-19 cells were treated with different concentrations of H_2_O_2_ (0–0.5 mM) for 4 h. (**C**) Effect of delphinidin against H_2_O_2_-induced cytotoxicity in ARPE-19 cells. ARPE-19 cells were pretreated with different concentrations of delphinidin (0–100 μg/ml) for 24 h and then treated with H_2_O_2_ (0.5 mM) for 4 h. All data were expressed as Mean ± SD of three experiments and each experiment included triplicate repeats. *P<0.05, **P<0.01 versus normal control group. ^#^*P*<0.05, ^##^*P*<0.01 versus H_2_O_2_ control group.

First, we examined the potential cytotoxicity of delphinidin on human ARPE-19 cells using MTT assay. As seen in Figure 2A, no change in cell viability occurred in cells treated with different concentrations of delphinidin ranging from 25 to 100 μg/ml. However, beyond this concentration, we would see a slight decrease in cell viability by delphinidin at 200 μg/ml, which was not significant from the untreated control (*P*>0.05). As such, the concentrations of delphinidin at 25, 50, and 100 μg/ml were used in the following assays. Then, in order to choose an appropriate concentration of H_2_O_2_ to test the protective effect of delphinidin on H_2_O_2_-incued cytotoxicity, the cell viability of ARPE-19 cells was examined after H_2_O_2_ (0–0.5 mM) exposure for 4 h. Figure 2B showed that cell viability decreased significantly from 100 ± 7.7% at untreated group to 78.34 ± 6.8% at 0.125 mM, 55.54 ± 4.7% at 0.25 mM, 25.78 ± 2.1% at 0.5 mM, respectively. Therefore, we chose a concentration of 0.5 mM H_2_O_2_ to be used as the working concentration in the following experiments.

Next, we aimed to evaluate whether delphinidin treatment could protect ARPE-19 cells from H_2_O_2_-induced cell death. ARPE-19 cells were pre-incubated in medium containing delphinidin (25, 50, and 100 μg/ml) for 12, 24, and 48 h, and then replaced with a fresh culture medium containing H_2_O_2_ (0.5 mM) for 4 h. The result showed that there was a slight protective effect on cell death induced by H_2_O_2_ after delphinidin (25, 50, and 100 μg/ml) treatment for 12 h, which is not statistically significant from the control group (*P*>0.05). Apparently, a notable protective effect can be observed after treatment with delphinidin at 24 h and its efficiency approached the protective effect of delphinidin at 48 h (*P*<0.05, data not shown). Therefore we only chose 24-h incubation time to assess the protective ability of delphinidin on cell viability of ARPE-19 cells following insult of H_2_O_2_. As demonstrated in Figure 2C, pre-treatment with 25, 50, and 100 μg/ml of delphinidin significantly raised the cell viability up to 62.1 ± 4.5, 77.68 ± 5.9, and 85.10 ± 6.8% with relative to that observed in control cells (*P*<0.05 or *P*<0.01).

### Delphinidin protects H_2_O_2_-induced apoptosis in ARPE-19 cells

**Figure 3:**
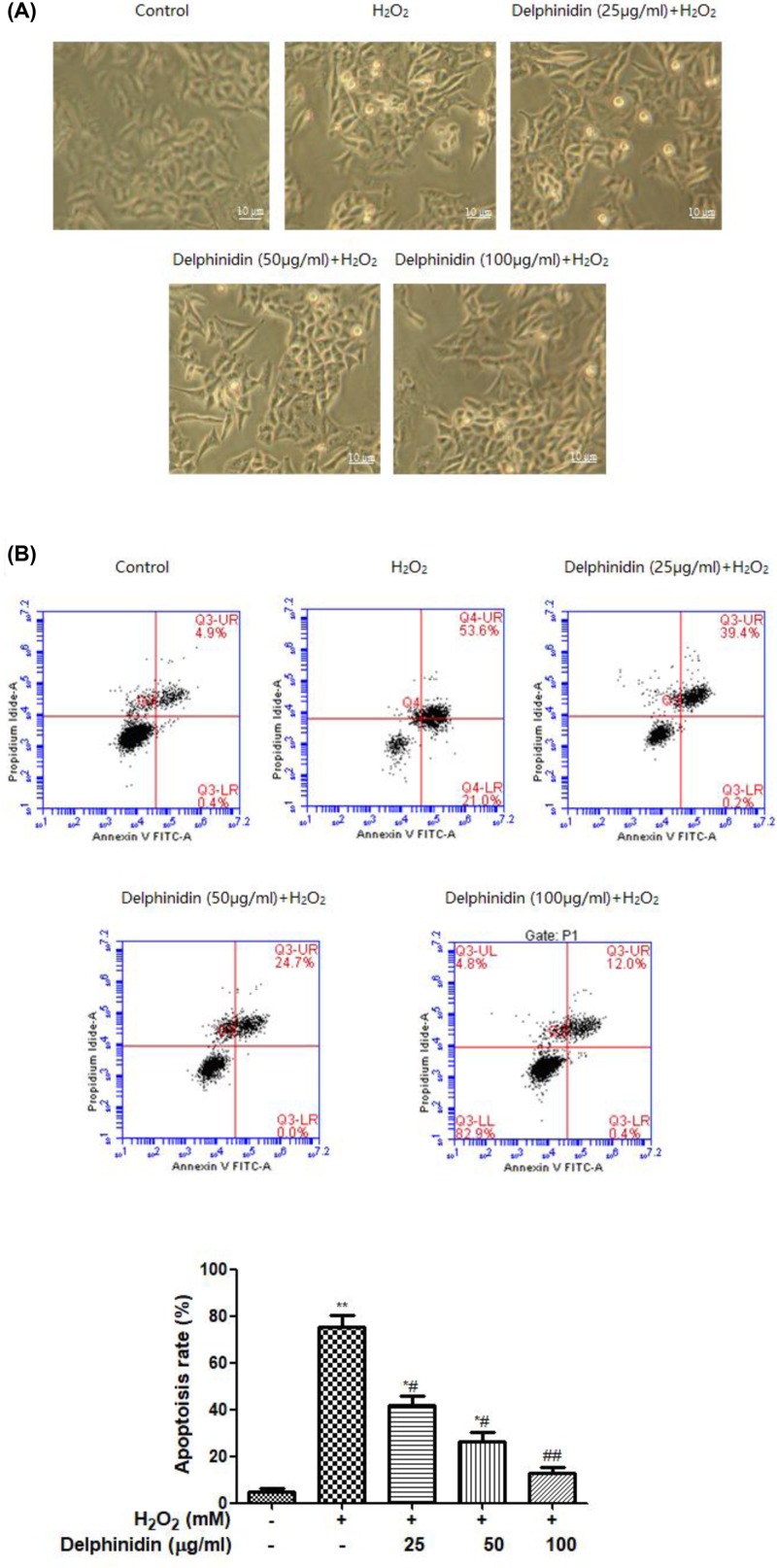
Delphinidin protects H_2_O_2_-induced apoptosis in ARPE-19 cells (**A**) Morphological images of ARPE-19 cells following different treatment. (**B**) Effect of delphinidin against H_2_O_2_-induced apoptosis in ARPE-19 cells. ARPE-19 cells were pretreated with different concentrations of delphinidin (0–100 μg/ml) for 24 h and then treated with H_2_O_2_ (0.5 mM) for 4 h. All data were expressed as Mean ± SD of three experiments and each experiment included triplicate repeats. *P<0.05, **P<0.01 versus normal control group. ^#^*P*<0.05, ^##^*P*<0.01 versus H_2_O_2_ control group.

To further investigate this protective effect of delphinidin on cell viability of ARPE-19 cells, the morphological change and apoptosis rate of ARPE-19 cells, incubated with delphinidin (25, 50, and 100 μg/ml) for 24 h and then exposed to 0.5 mM H_2_O_2_ for 4 h, were determined under a phase contrast microscope and flow cytometry (Figure 3A,B), respectively. Consistently, exposure to 0.5 mM H_2_O_2_ for 4 h led to a significantly higher rate of total apoptosis (combined early- and late-apoptotic cells: 75.34 ± 5.54%) compared with that of the control group (total apoptotic rate: 4.85 ± 0.38%, *P*<0.01); but pre-treatment of ARPE-19 cells with delphinidin at the concentration of 25, 50, and 100 μg/ml before H_2_O_2_ exposure significantly decreased 44.57 ± 3.85, 65.85 ± 5.54, and 83.34 ± 6.89% of H_2_O_2_-induced cell apoptosis, respectively (*P*<0.05, *P*<0.01 vs the oxidative model).

### Delphinidin regulates H_2_O_2_-induced apoptosis-related protein expression in ARPE-19 cells

**Figure 4:**
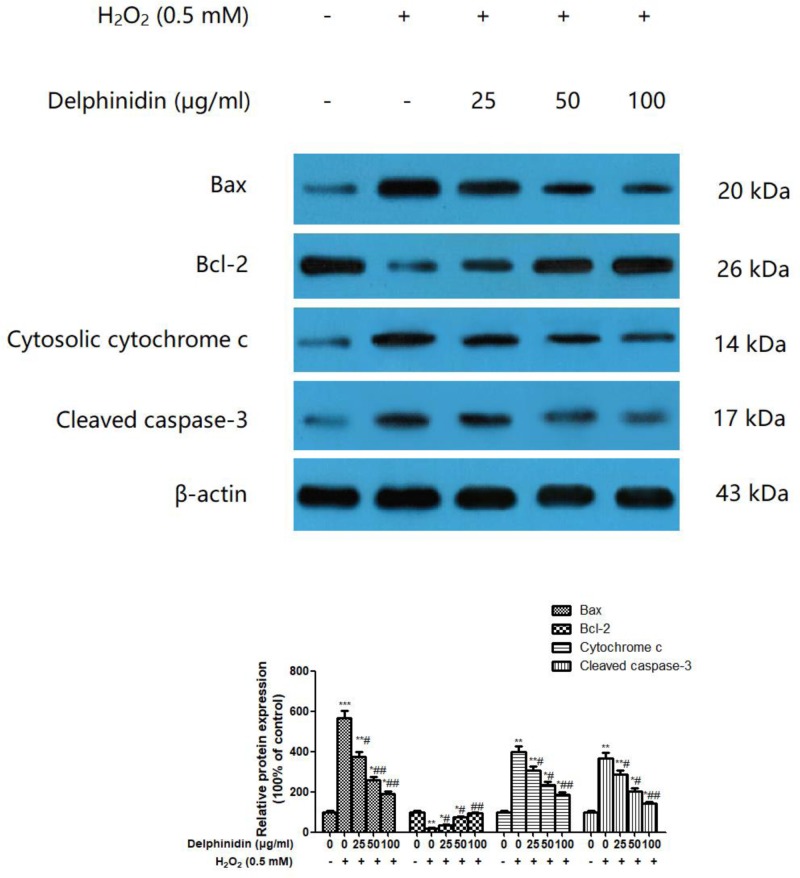
Effect of delphinidin (25, 50, and 100 μg/ml) on the protein expression of Bax, Bcl-2, cytochrome *c*, and cleaved caspase-3 in H_2_O_2_ post-treated ARPE-19 cells assessed by Western blot ARPE-19 cells were pretreated with different concentrations of delphinidin (0–100 μg/ml) for 24 h and then treated with H_2_O_2_ (0.5 mM) for 4 h. All data were expressed as Mean ± SD of three experiments and each experiment included triplicate repeats. *P<0.05, **P<0.01, ***P<0.001 versus normal control group. ^#^*P*<0.05, ^##^*P*<0.01 versus H_2_O_2_ control group.

**Figure 5:**
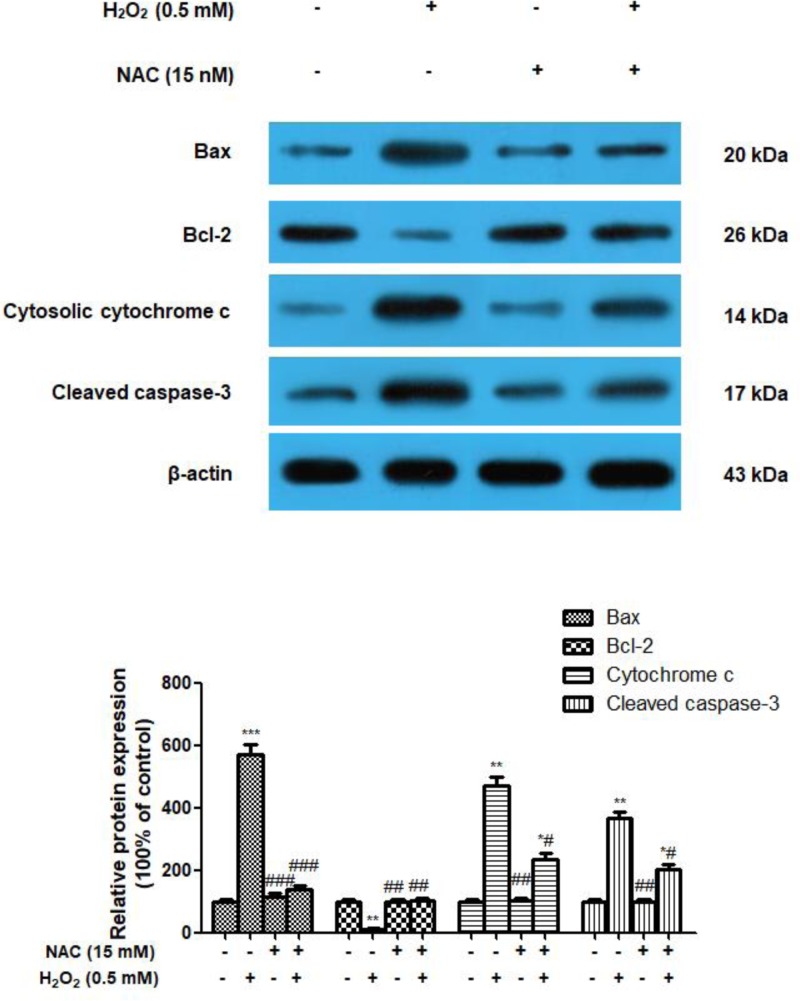
Effect of NAC (15 mM) on the protein expression of Bax, Bcl-2, cytochrome *c* and cleaved caspase-3 in H_2_O_2_ post-treated ARPE-19 cells assessed by Western blot ARPE-19 cells were pretreated with NAC (15 mM) for 24 h and then treated with H_2_O_2_ (0.5 mM) for 4 h. All data were expressed as Mean ± SD of three experiments and each experiment included triplicate repeats. *P<0.05, **P<0.01, ***P<0.001 versus normal control group. ^#^*P*<0.05, ^##^*P*<0.01 versus H_2_O_2_ control group.

In view of protective effect of delphinidin against H_2_O_2_-induced apoptosis in ARPE-19 cells, we further investigated the effects of delphinidin on the expression of apoptosis-related molecules, i.e. Bax, Bcl-2, cytochrome *c*, and cleaved caspase-3, by Western blotting (Figure 4). Compared with normal control cells, exposure to 0.5 mM H_2_O_2_ induced lower level of Bcl-2 and higher levels of Bax, cytochrome *c*, and caspase-3, which was consistent with our flow cytometry results. However, pre-treatment of ARPE-19 cells with delphinidin (25, 50, and 100 μg/ml) for 24 h dose-dependently reversed this situation, as shown by the decreased expression of Bax, cytochrome *c*, and caspase-3, as well as the increased expression of Bcl-2, which were statistically significant from those in H_2_O_2_ group (*P*<0.05 or *P*<0.01). The same tendency was observed in ARPE-19 cells pre-treated with 15 mM of NAC for 24 h before the addition of H_2_O_2_ (Figure 5).

### Delphinidin reduces H_2_O_2_-induced intracellular ROS production and Nox1 protein expression in ARPE-19 cells

**Figure 6:**
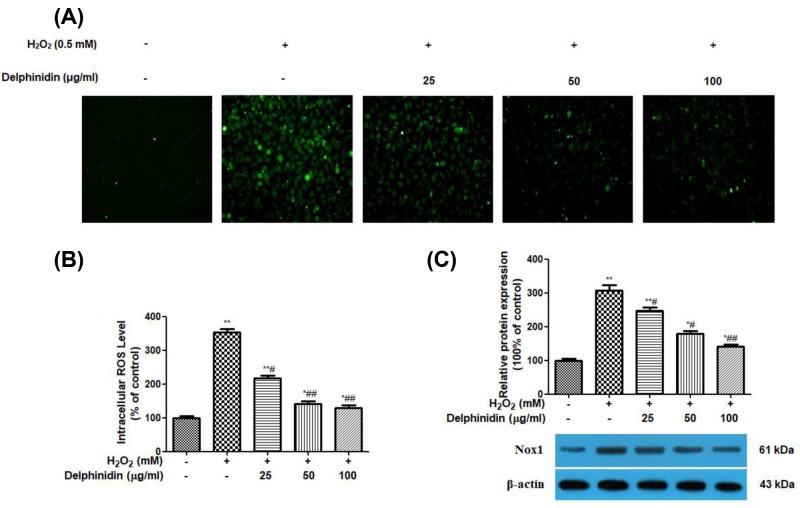
Delphinidin reduces H_2_O_2_-induced intracellular ROS production and Nox1 protein expression in ARPE-19 cells (**A**) Effect of delphinidin (25, 50, and 100 μg/ml) on oxidative stress in H_2_O_2_ post-treated ARPE-19 cells assessed by DCFH-DA staining. (**B**) Quantitative result of DCFH-DA staining by flow cytometric assay. (**C**) Effect of delphinidin (25, 50, and 100 μg/ml) on the protein expression of Nox1 in H_2_O_2_ post-treated ARPE-19 cells assessed by Western blot. ARPE-19 cells were pretreated with different concentrations of delphinidin (0–100 μg/ml) for 24 h and then treated with H_2_O_2_ (0.5 mM) for 4 h. All data were expressed as Mean ± SD of three experiments and each experiment included triplicate repeats. *P<0.05, **P<0.01, versus normal control group. ^#^*P*<0.05, ^##^*P*<0.01 versus H_2_O_2_ control group.

To detect potential ROS scavenging capabilities of delphinidin against H_2_O_2_, a DCFH-DA reagent staining was performed to quantify the ROS levels in delphinidin-pretreated ARPE-19 cells. The higher the fluorescence intensity, the more ROS remains, and *vice versa*. As shown in Figure 6A,B, the DCF fluorescence intensity increased significantly in H_2_O_2_-treated cells when compared with that in the normal control cells where no H_2_O_2_ was added (*P*<0.01). However, addition of delphinidin (25, 50, and 100 μg/ml) for 24 h gradually reduced the DCF fluorescence level and almost completely suppressed it at a concentration of 100 μg/ml (*P*<0.01). In addition, delphinidin pre-treatment attenuated the expression of Nox1 protein induced by H_2_O_2_ in a dose-dependent manner and significant statistical difference was observed compared with H_2_O_2_ control group (*P*<0.05 or *P*<0.01, Figure 6C).

### Delphinidin improves H_2_O_2_-induced decreasing antioxidant status in ARPE-19 cells

**Figure 7:**
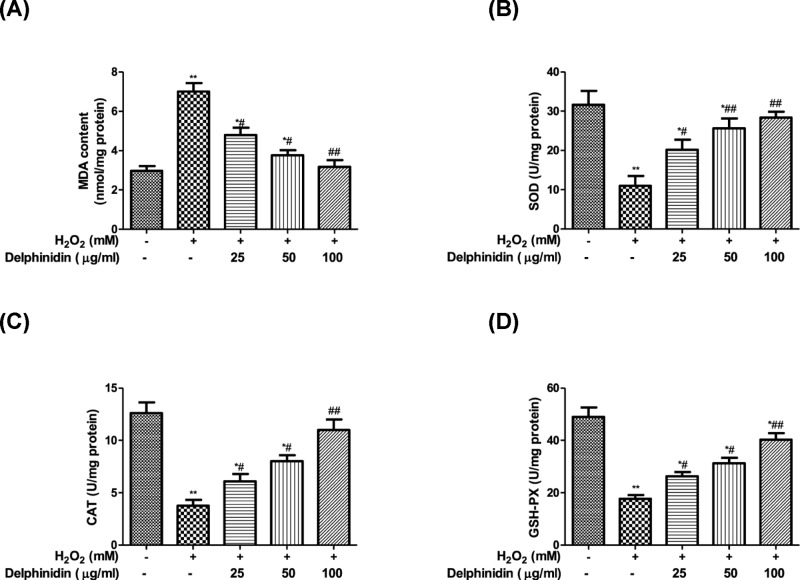
Delphinidin improves H_2_O_2_-induced decreasing antioxidant status in ARPE-19 cells (**A**) Effect of delphinidin (25, 50, and 100 μg/ml) on MDA level in H_2_O_2_ post-treated ARPE-19 cells assessed by colorimetric assay kit. (**B**) Effect of delphinidin (25, 50, and 100 μg/ml) on SOD activities in H_2_O_2_ post-treated ARPE-19 cells assessed by colorimetric assay kit. (**C**) Effect of delphinidin (25, 50, and 100 μg/ml) on CAT activities in H_2_O_2_ post-treated ARPE-19 cells assessed by colorimetric assay kit. (**D**) Effect of delphinidin (25, 50, and 100 μg/ml) on GSH-PX activities in H_2_O_2_ post-treated ARPE-19 cells assessed by colorimetric assay kit. ARPE-19 cells were pretreated with different concentrations of delphinidin (0–100 μg/ml) for 24 h and then treated with H_2_O_2_ (0.5 mM) for 4 h. All data were expressed as Mean ± SD of three experiments and each experiment included triplicate repeats. *P<0.05, **P<0.01, versus normal control group. ^#^*P*<0.05, ^##^*P*<0.01 versus H_2_O_2_ control group.

Given that oxidative stress plays a vital role in the aging status of RPE cells, we next measured the activities of three oxidative stress biomarkers (SOD, CAT, and GSH-PX) and lipid oxidation indicator MDA level in cultured ARPE-19 cells following different treatment. As shown in Figure 7A–D, co-culture of ARPE-19 cells with H_2_O_2_ for 4 h resulted in a higher MDA level, but lower activities of SOD, CAT, and GSH-PX than the untreated group (*P*<0.05). Delphinidin treatment alone did not have any effect on the MDA level and activities of SOD, CAT, and GSH-PX (data not shown), however in oxidative stress conditions, the increased level of MDA and decreased activities of SOD, CAT, and GSH-PX in ARPE-19 cells cultured with H_2_O_2_ were abrogated by the addition of delphinidin (25, 50, and 100 μg/ml) in a concentration-dependent manner.

**Figure 8:**
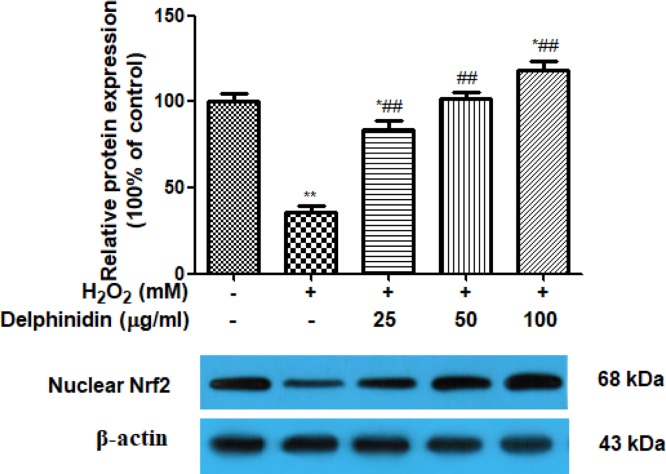
Effect of delphinidin (25, 50, and 100 μg/ml) on the protein expression of nuclear Nfr2 in H_2_O_2_ post-treated ARPE-19 cells assessed by Western blot ARPE-19 cells were pretreated with different concentrations of delphinidin (0–100 μg/ml) for 24 h and then treated with H_2_O_2_ (0.5 mM) for 4 h. All data were expressed as Mean ± SD of three experiments and each experiment included triplicate repeats. *P<0.05, **P<0.01, versus normal control group. ^##^*P*<0.01 versus H_2_O_2_ control group.

To gain insight into the mechanisms underlying the improved intracellular redox state by delphinidin in this model, we further analyzed the effect of delphinidin on the expression of Nrf2 translocation in ARPE-19 cells, a master regulator of cellular antioxidant response. As shown in Figure 8, the Nrf2 protein expression in nuclear fractions was increased by treatment with delphinidin in a concentration-dependent manner.

## Discussion and conclusions

A growing body of evidence supports the idea that ROS-induced oxidative stress can cause damages to the retinal endothelial cells, eventually leading to retinal degenerative diseases, such as AMD [23,24]. Oxidative stress mainly results from a serious imbalance between the ROS generation and antioxidant defenses, which would lead to less photoreceptor cell repair and regeneration with increasing age because of accumulation of abnormal proteins and lipids [25,26]. Thus, a balanced redox state is crucial for delaying the progression of AMD and vision loss.

More importantly, data from clinical and experimental studies suggested that daily dietary supplement of natural antioxidants, such as β-carotenoid, lutein, zeaxanthin, and anthocyanins, could be a potential strategy for maintenance of lens opacity and retinal function [27]. Especially, several lines of evidence demonstrated the protective effect of anthocyanidins toward UV or visible light-induced retinal damage [28,29]. Delphinidin, a representative anthocyanidin (aglycon of anthocyanin), is prevalent in vegetables and fruits and exerts the strongest antioxidative efficiency among six types of anthocyanidins in the human diet [30]. Although delphinidin is a major bioactive component of anthocyanidin, the mechanisms responsible for its effects against H_2_O_2_-induced oxidative stress remain poorly defined. Therefore, the purpose of the present study was to examine the potential protective effects of delphinidin against retinal endothelial cells death induced by H_2_O_2_.

RPE cells lie behind the photoreceptor cells and have the most common oxidative-damaged structure in the retina. H_2_O_2_ is a major factor implicated in inducing oxidative damage and cell death in different types of cells, including retinal cells [13]. Here, in the present study, H_2_O_2_ was used to challenge ARPE-19 cells to induce oxidative stress and cell cytotoxicity to mimic the pathogenesis of AMD. As assayed by MTT assay and flow cytometry, the exposure to 0.5 mM H_2_O_2_ decreased cell viability of ARPE-19 cells by 74.2% and induced apoptosis by ∼75.34%, whereas pretreatment of ARPE-19 cells with delphinidin (25, 50, and 100 μg/ml) significantly increased cell viability and reduced the apoptosis from oxidative stress. Intriguingly, delphinidin pretreatment alone has no effect on the cell growth and apoptosis of ARPE-19 cells.

Numerous studies have shown that ROS-triggered apoptotic pathway activation plays a crucial role in the pathogenesis of AMD [31]. It has been reported that compounds which improve mitochondrial functions may show beneficial effects in preventing AMD [32]. Bcl-2 family proteins are well-known key regulators in regulating cell apoptosis and include anti-apoptotic (e.g. Bcl-2) and pro-apoptotic (e.g. Bax) protein [33]. Evidence indicates that an increase in Bax/Bcl-2 ratio would change the permeability of the mitochondrial membranes, which also results in the release of cytochrome *c* and the subsequent activation of caspases [34,35]. Among them, cleaved-caspase-3 serves as the key executioner in cell death through receptor-mediated or mitochondria dependent-induced apoptosis [36]. The present study demonstrated that H_2_O_2_ exposure led to an increase in Bax, cytosolic cytochrome *c*, and cleaved caspase-3 protein expression, but a lower expression of Bcl-2 protein, compared with the control groups. However, pretreatment of cells with delphinidin (25, 50, and 100 μg/ml) for 24 h before exposure to H_2_O_2_ effectively reverse this change, as evidenced by the decreased protein expression of Bax, cytosolic cytochrome *c* and cleaved caspase-3, along with an augment of Bcl-2 protein. Furthermore, NAC, a direct scavenger of ROS, significantly reversed the H_2_O_2_-induced change of anti-apoptotic and pro-apoptotic protein expression in ARPE-19 cells. This result suggested that intracellular ROS generation was related to H_2_O_2_-induced apoptosis in ARPE-19 cells. Thus, delphinidin protected RPE cells against H_2_O_2_-induced partly by regulating caspase-3-mediated apoptotic pathway.

Recent studies have suggested that apoptosis of epithelial cells is associated with the production of multiple ROS, and the inhibition of ROS by antioxidant enzymes effectively inhibits apoptotic events [37]. ROS generation in cells occurs with mitochondria, peroxisomes, plasma membrane NADPH oxidase (NOX), and other cellular elements as well [38]. Among different sources of ROS, high glucose-induced enhanced ROS generation is primarily mediated by mitochondria and NOX [39]. Moreover, NOX1 has also been reported as a main contributor of increased ROS generation in the human retinal pigment epithelium cells [40]. In this case, the production of ROS and antioxidant enzymes (SOD, CAT, and GSH-PX) activities, as well as MDA level and Nox1 protein expression, were investigated in this study. As anticipated, H_2_O_2_ stimulation increased the production of ROS and Nox1 protein expression and this change was reversed by the addition of delphinidin, indicating inhibition of Nox1/ROS system attributed to the protective effect of delphinidin against H_2_O_2_ toxicity in ARPE-19 cells. Furthermore, the pre-treatment of delphinidin markedly reduced H_2_O_2_-induced MDA generation and increased the activities of SOD, CAT, and GSH-PX suppressed by H_2_O_2_ in ARPE-19 cells. Of note, intracellular redox state, as determined by the balance between ROS and antioxidant defense mechanisms, is a key determinant of programmed cell death. The transcription factor Nrf2 is crucial for the efficient detoxification of reactive metabolites and ROS [41]. The majority of genes encoding xenobiotic detoxifying and antioxidant enzymes have an enhancer region containing the antioxidant response element (ARE) [42]. Nrf2 is a critical transcriptional activator that binds to ARE and increased transcription of a number of cytoprotective genes in various cell types [43,44]. Therefore, we further examine the effects of delphinidin on Nrf2 translocation in ARPE-19 cells. Western blots of nuclear fractions showed that delphinidin treatment caused an increase in the abundance of nuclear Nrf2. Thus, these findings suggested that delphinidin has potential to protect RPE cells from H_2_O_2_-induced oxidative stress, which was achieved by suppressing ROS generation in Nox1-dependent manner and MDA level, and enhancing antioxidant enzymes activities of SOD, CAT, and GSH-PX through up-regulation of the nuclear Nrf2 protein.

Additionally, our preliminary data showed that delphinidin mainly exerted its activity via triggering NOX1-mediated activation of AMP-activated protein kinase (AMPK). AMPK inhibition significantly reversed the protective effect of delphinidin against H_2_O_2_ toxicity in ARPE-19 cells (data not shown). Also, AMPK knockdown reversed the effect of delphinidin against H_2_O_2_-induced ROS production, mitochondrial dysfunction, increased pro-apoptotic protein expression of cytochrome *c*, caspase-3, and Bcl-2, as well as decreased anti-apoptotic Bax protein expression. AMPK has been identified as a potential therapeutic target for many human diseases and its activation is also involved in the prevention of various retinal degenerative diseases [45,46]. Therefore, activating NOX1-mediated AMPK signaling pathway is likely to be a novel approach in the treatment of AMD, which would be initiated in depth in the oncoming work.

Taken together, the present study provided evidence that delphinidin effectively protected human RPE cells from H_2_O_2_-induced oxidative damage, at least partly, via anti-apoptotic and antioxidant effects. These findings shed light on the pharmacological application of delphinidin in preventing eye diseases such as AMD and further investigations, including animal study, are required to better reveal the mechanisms in detail before its clinical application.

## Abbreviations

AMD: age-related macular degeneration
AMPK: AMP-activated protein kinase
ARE: antioxidant response element
CAT: catalase
DCF: 2′,7′-dichlorodihydrofluorescein
DCFH-DA: 2′,7′-dichlorofluorescin diacetate
Delphinidin: 2-(3,4,5-trihydroxyphenyl) chromenylium-3,5,7-triol
DMEM: Dulbecco’s Modified Eagle’s Medium
DMSO: dimethylsulfoxide
FITC: fluorescein isothiocyanate
GSH-PX: glutathione-peroxidase
H_2_O_2_: hydrogen peroxide
MDA: malondialdehyde
MTT: 3-(4,5-dimethylthiazol-2-yl)-2,5-diphenyltetrazolium bromide
NAC: N-acetylcysteine
NADPH: nicotinamide adenine dinucleotide phosphate
NOX: NADPH oxidase
PI: propidium iodide
ROS: reactive oxygen species
SOD: superoxide dismutase
